# A Prospective, Observational Cost Comparison of Laparoscopic and Open Appendicectomy in Three Tertiary Hospitals in Nigeria

**DOI:** 10.1007/s00268-023-07148-5

**Published:** 2023-10-11

**Authors:** Adewale Adisa, Mwayi Kachapila, Christopher Ekwunife, Felix Alakaloko, Balogun Olanrewaju, Bryar Kadir, Dmitri Nepogodiev, Adewale Aderounmu, Innocent Igwilo, Omar Omar, Raymond Oppong, Joana Simoes, Aneel Bhangu, Adesoji Ademuyiwa, the NIHR Global Health Research Unit on Global Surgery

**Affiliations:** 1 Department of Surgery, Obafemi Awolowo University Teaching Hospitals Complex, Ile-Ife, 220005 Nigeria; 2 NIHR Global Health Research Unit on Global Surgery, University of Birmingham, Birmingham, United Kingdom; 3 Health Economics Unit, University of Birmingham, Birmingham, United Kingdom; 4 Department of Surgery, Federal Medical Center, Owerri, Nigeria; 5 Department of Surgery, Lagos University Teaching Hospital, Lagos, Nigeria

## Abstract

**Background:**

The clinical benefits of laparoscopic appendicectomy are well recognized over open appendicectomy. However, laparoscopic procedures are not frequently conducted in many low-and middle-income countries (LMICs) for several reasons, including perceived higher costs. The aim of this study was to assess the feasibility and cost of laparoscopic appendicectomy compared to open appendicectomy in Nigeria.

**Methods:**

A multicenter, prospective, cohort study among patients undergoing appendicectomy was conducted at three tertiary hospitals in Nigeria. Data were collected from October 2020 to February 2022 and analyses compared the average healthcare costs at 30 days after surgery. Quantile regression was conducted to identify variables that had an impact on the costs, reported in Nigerian Naira (Naira) and US dollars ($), with standard deviations (SD).

**Findings:**

This study included 105 patients, of which 39 had laparoscopic appendicectomy and 66 had open appendicectomy. The average healthcare cost of laparoscopic appendicectomy (147,562 Naira (SD: 97,130) or $355 (SD: 234)) was higher than open appendicectomy (113,556 Naira (SD: 88,559) or $273 (SD: 213)). The average time for return to work was shorter with laparoscopic than open appendicectomy (mean: 8 days vs. 14 days). At the average daily income of $5.06, laparoscopic appendicectomy was associated with 9778 Naira or $24 cost savings in return to work. Further, 5.1% of laparoscopic appendicectomy patients had surgical site infections compared to 22.7% for open appendicectomy. Regression analysis results showed that laparoscopic appendicectomy was associated with $14 higher costs than open appendicectomy, albeit non-significant (*p* = 0.53).

**Interpretation:**

Despite selection bias in this real-world study, laparoscopic appendicectomy was associated with a slightly higher overall cost, a lower societal cost, a lower infection rate, and a faster return to work, compared to open appendicectomy. It is technically and financially feasible, and its provision in Nigeria should be expanded.

**Supplementary Information:**

The online version contains supplementary material available at 10.1007/s00268-023-07148-5.

## Introduction

The clinical benefits of laparoscopic surgery over open surgery are well recognized. These include reduced postoperative pain, shorter hospital length of stay, earlier return to normal activities, and lower rates of surgical site infection [1–3]. Laparoscopy has become the standard of care in many centers in high-income countries (HICs). Surgeons practicing in low- and middle-income countries (LMICs) have argued that it is even more beneficial for poor patients to access laparoscopy, as the advantages may be more pronounced in low resource settings, where social support systems are less developed and patients are at higher risk of catastrophic healthcare spending [4, 5].

Adoption of laparoscopic surgery in LMICs remains low, creating a global disparity in surgical care [6]. Reasons for these include high cost of initial set-up, maintenance of equipment, the need for ongoing training of surgical teams, and lack of regular consumables [4–8]. Cost remains one of the strongest barriers perceived by surgeons in LMICs against the adoption of the technique [6–9]. Most studies comparing the cost of open versus laparoscopic surgeries have been carried out in HICs where healthcare financial systems are different from LMICs [10, 11]. Only a few studies have highlighted the cost implications of laparoscopic surgeries in LMICs. Lombardo et al. [12] examined the cost and outcomes of open versus laparoscopic cholecystectomies in some rural and urban hospitals in Mongolia, concluding that laparoscopy attracted a higher cost, but the difference was not statistically significant. However, only the direct costs to the patients and insurance were analyzed in their study.

The aim of the present study was to evaluate the cost of laparoscopic (LA) versus open appendicectomy (OA) for uncomplicated acute appendicitis at three hospitals in Nigeria. Acute appendicitis was selected as the disease of interest for uniformity and ease of comparison between surgical approaches. By doing so in detail, we aimed to better understand how to address barriers that may limit further expansion of laparoscopic surgery in Nigeria and other LMICs.

## Methods

### Study setting

This was a multicenter, prospective, cohort study of consecutive children and adults undergoing elective or emergency appendicectomy for uncomplicated acute appendicitis via conventional open or laparoscopic approach in three selected hospitals in Nigeria.

The three hospitals were Obafemi Awolowo University Teaching Hospitals Complex, Ile-Ife, (a semi-urban location in the southwestern region), Federal Medical Center, Owerri, (one of the largest urban cities in the southeast), and Lagos University Teaching Hospital, Lagos, (the commercial hub of the country). The three are publicly funded, government-owned tertiary referral hospitals, selected because they have personnel and equipment setup for laparoscopy [Supplementary Fig. 1]. The study was approved by the Ethical Review Committee of each hospital.

### Patient and technique selection

All patients with a diagnosis of acute appendicitis had an abdominal ultrasound performed to determine eligibility for inclusion. Uncomplicated acute appendicitis was defined as one with no clinical or radiological findings suggestive of abscess, gangrene, peritonitis, or mass formation. Those with clinical findings suggestive of complicated acute appendicitis were excluded. All patients were consented to participate in the study during their preoperative assessment. The choice of laparoscopic or open approach to surgery was left to the discretion of the consultant surgeon, with the reason for the choice as well as reasons for any conversions of laparoscopy to open, were documented.

### Resource use, unit costs and patient follow-up

This study collected data from a healthcare payer perspective, quantifying costs to only the healthcare sector [13, Supplementary Table 1]. The resource use data were collected from patients using case report forms (CRFs, online Appendix 1) administered during the study. Hospital unit costs were collected using unit cost forms completed at hospital level. Postoperative data collection was done in-person or via telephone on postoperative day 30. Surgical site infection was defined and graded according to the center for disease control (CDC) criteria. All the data were concurrently collected at the three participating sites from May 2020 to February 2022, and despite clinical service interruptions during the COVID-19 pandemic, consecutive cases were still included.

### Primary analysis

The analysis was conducted from an intention-to-treat approach, therefore, all patients who were recruited into the study were included in the analysis. The primary outcome measure was the average healthcare cost for both LA and OA groups up to 30-days postoperatively. These costs included the cost of surgery, hospital admission (hospital stay), diagnostics, post-discharge healthcare, reoperation, and readmission and the costs were estimated using the bottom-up approach (identified all resource use and multiplied them with the unit cost) [14]. The cost of instruments used for open surgery, laparoscopic tower, and anesthetic machine was annuitized assuming 20-year useful life, assumed to have a zero-resale value and were discounted at a 3% discount rate, a rate that is commonly used and recommended for economic evaluations in LMICs [15].

### Postoperative costs estimation

We used data gathered from a sub-study with the FALCON trial that recruited consecutive patients from India, Nigeria, Mexico, and Ghana to collect direct postoperative healthcare costs including wound dressing, healthcare consultations, transport, and indirect costs of lost income to the patients that their families [Supplementary Table 1]. Based on this evidence, the postoperative care costs of patients with an SSI in the current study were increased by 75.5% to include the impact of SSI treatment on the total costs.

### Productivity costs

Societal costs, which are all the costs of an intervention including costs incurred outside the healthcare systems were assessed [15]. The societal costs (indirect costs) were measured as the lost income between the day of surgery and the day the patient returned to work. The Human Capital Approach was used to value time lost before returning to work by multiplying the number of days taken to return to work and the average daily earnings in Nigeria. The 2019 monthly average earnings published by the International Labor Organization (ILO) [16], were multiplied by 12, then divided by 365 to get the average daily income.

Sensitivity analysis was conducted to assess if there could have been substantial changes on the primary analysis results (base case results) when the values of given input parameters changed. Useful life of capital items was increased from 20 to 40 years and the discount rate was increased from 3 to 5%.

### Statistical analysis

Data cleaning and statistical analysis were conducted using STATA, version 17.0 (StataCorp LLC; College Station, TX, USA). The patient characteristics, operative and clinical outcomes data were presented using percentages, median, and inter-quartile range (IQR). Variables used in cost estimation were presented using mean and standard deviation (SD) because mean costs are more meaningful to decision makers than median costs [17]. All costs in Naira were inflated to 2022 Naira and then converted to US dollars ($) using the official inflation and exchange rates published by the Central Bank of Nigeria [18, 19]. Normality assumptions of the residuals were assessed using histograms and normality plot. As the cost data were skewed, quantile regression analysis was conducted to identify variables that had an impact on the costs. The dependent variable was the average cost of treatment. The pre-specified independent variables were surgical approach, sex, age, employment status, and histology outcome. A *p* value of <0.05 was statistically significant.

## Results

### Patient characteristics

This study included 105 patients who had appendectomies at three hospitals in Nigeria between May 2020 and February 2022. Of these, 39 had LA and 66 had OA. Baseline patient characteristics between the two groups are presented in Table 1. The median age of the laparoscopic appendicectomy patients was 19 years (inter-quartile range (IQR): 11, 24) compared to 18 years (IQR: 12, 25) for OA. Twenty out of 39 patients in the LA group were females compared to 33 out of 50 patients in the OA group. The average distance to the hospital was 21.3 km (SD: 49.2) in the LA group compared to 10.2 km (SD: 11.6) in the OA group.

**Table 1:** Demographic and preoperative data by operative approach

	Laparoscopic appendicectomy (*n* = 39)	Open appendicectomy (*n* = 66)	*p* value
Age in years			0.868
Median (IQR)	19 (11, 29)	18 (12, 25)	
Min–Max	5–63	7–51	
Monthly income (Naira)			0.605
Median (IQR)	0 (0, 35,000)	0 (0.20000)	
Min–Max	0–400,000	0–3,000,000	
Missing	10	13	
Monthly income ($)			0.605
Median (IQR)	0 (0, 84)	0 (0. 48)	
Min–Max	0–963	0–7220	
Missing	10	13	
Distance to hospital in km			0.894
Median (IQR)	3 (5, 13)	3 (5, 12)	
Min–Max	1–196	1–125	
Distance in km (mean, sd)	21.3 (49.2)	10.2 (11.6)	0.05
Sex, *n* (%)			0.999
Female	20 (51.3)	33 (50.0)	
Male	19 (48.7)	33 (50.0)	
Highest level of education attended, *n* (%)			0.868
No-formal education	0 (0)	0 (0)	
Primary school	9 (23.1)	13 (9.7)	
Secondary school	12 (30.8)	24 (36.4)	
Post-secondary school	18 (46.1)	29 (43.9)	
Occupation, *n* (%)			0.825
Formal employment	8 (20.5)	10 (15.1)	
Self-employment	2 (5.1)	4 (6.1)	
Student	27 (69.2)	50 (75.8)	
Unemployed	2 (5.1)	2 (3.0)	

IQR stands for inter-quartile range

### Intraoperative characteristics

The intraoperative data in Table 2 show that for some parameters there were significant differences in hospital resource use between the two groups. The median number of swab packs used in the LA group was 2 (IQR: 2, 3) compared to 4 (IQR: 2, 5) in the OA group. In the LA group only 31% needed inpatient dressings compared to 56% in the OA group. Eighty percent of patients in the LA group needed wound infiltration with local anesthesia compared 36% of patients in the OA group and 97% of the operations in the laparoscopic group were done by consultant surgeons compared to 30% in the OA group. OA was mainly opted for because of unavailability of a surgeon trained in laparoscopic surgery (65%) or lack of supplies for laparoscopic surgery (11%) while laparoscopic surgery was mainly chosen because it was the preference of the available surgeon (72%) or patient (21%).

**Table 2:** Intraoperative outcomes in patients undergoing appendicectomy

	Laparoscopic appendicectomy (*n* = 39)	Open appendicectomy (*n* = 66)	*p* value
Operation time in minutes			0.955
Median (IQR)	55 (45, 75)	56 (45, 70)	
Min–Max	30–120	30–122	
Operation time in minutes (mean, sd)	72.1 (24.5)	62.8 (22.5)	0.22
Reason for choice of operation			<0.001
Emergency	0 (0)	5 (7.6)	
Hospital protocol	2 (5.1)	0 (0)	
Laparoscopic supplies not available	0 (0)	7 (10.6)	
Laparoscopic surgeon not available	0 (0)	43 (65.2)	
Patient condition	1 (2.6)	2 (3.0)	
Patient preference	8 (20.5)	1 (1.5)	
Surgeon preference	28 (71.8)	6 (9.1)	
Other	0 (0)	2 (3.0)	
Number of swab packs			<0.001
Median (IQR)	2 (2, 3)	4 (2, 5)	
Min–Max	1, 17	1, 15	
Number of swab packs used (mean, sd)	4 (3.85)	4 (1.92)	0.13
Lead surgeon, *n* (%)			<0.001
Consultant surgeon	38 (97.4)	20 (30.3)	
Registrar or trainee	1 (2.6)	46 (69.7)	
Clavien–Dindo grade, *n* (%)			0.825
0	7 (18.0)	3 (4.6)	
1	2 (5.1)	14 (21.1)	
2	0 (0)	0 (0)	
3	0 (0)	1 (1.5)	
4	0 (0)	0 (0)	
5	0 (0)	0 (0)	
Missing	30	48	
Antibiotics used, *n* (%)			–
Yes	39 (100)	66 (100.0)	
No	0 (0)	0 (0)	
Urinary catheter used, *n* (%)			0.672
Yes	27 (69.2)	42 (63.6)	
No	12 (30.8)	24 (36.4)	
Wound infiltration with local anaesthesia, *n* (%)			<0.001
Yes	31 (79.5)	24 (36.4)	
No	8 (20.5)	42 (63.6)	
X-ray conducted, *n* (%)			0.674
Yes	24 (61.5)	44 (66.7)	
No	15 (38.5)	22 (33.3)	
Inpatient dressings used, *n* (%)			0.015
Yes	12 (30.8)	37 (56.1)	
No	27 (69.2)	29 (43.9)	
CRP test conducted, *n* (%)			0.649
Yes	1 (2.6)	4 (6.1)	
No	38 (97.4)	62 (93.9)	
WCC blood test conducted, *n* (%)			0.254
Yes	38 (97.4)	60 (90.9)	
No	1 (2.6)	6 (9.1)	
LFT blood test conducted, *n* (%)			–
Yes	0 (0)	0 (0)	
No	39 (100)	66 (100)	
U&Es blood test conducted, *n* (%)			0.681
Yes	23 (59.0)	42 (63.6)	
No	16 (41.0)	24 (36.4)	

IQR stands for inter-quartile range

### Postoperative outcomes

There were significant differences in favor of LA with respect to clinical outcomes, (Table 3). LA patients had 2 days of hospital stay (SD: [2]) compared to 9 days (SD: 45) in the OA group, LA was associated with less SSIs (5%) compared to 23% observed in the open appendicectomy group. LA group returned to normal activities earlier, 8 days (SD: 3) compared to 14 days (SD: 5) in the OA group and LA patients also returned to work earlier, 12 days (SD: 5), compared to OA patients, 16 (SD: 6).

**Table 3:** Clinical outcomes

	Laparoscopic appendicectomy (*n* = 39)	Open appendicectomy (*n* = 66)	*p* value
Length of stay in days,			<0.001
Median (IQR)	2 (1, 2)	3 (2, 5)	
Min–Max	1–7	0–37	
Length of stay in days, (mean, SD)	2 (2)	9 (45)	<0.001
Appendix histology, *n* (%)			0.411
Normal appendix	1 (2.6)	1 (1.5)	
Simple appendix	22 (56.4)	44 (66.7)	
Complex appendix	14 (35.9)	17 (25.8)	
Histology not done	2 (5.1)	4 (6.1)	
SSI, *n* (%)			0.014
Yes	2 (5.1)	15 (22.7)	
No	37 (94.9)	51 (77.3)	
Readmission, *n* (%)			0.393
Yes	0 (0)	2 (3.0)	
No	39 (100)	64 (97.0)	
Reoperation, *n* (%)			0.393
Yes	0 (0)	2 (3.0)	
No	39 (100)	64 (97.0)	
Returned to normal activities, *n* (%)			0.057
Yes, completely	0 (0)	1 (1.5)	
Some, but not all	37 (94.9)	52 (78.8)	
Not at all	2 (5.1)	13 (19.7)	
Days to return to normal activities			<0.001
Median (IQR)	7 (6, 10)	14 (11, 15)	
Min–Max	3, 14	5, 25	
Days to return to normal activities, (mean, SD)	8 (3)	14 (5)	<0.001
Returned to work, *n* (%)			0.017
Yes	30 (76.9)	43 (65.1)	
No	1 (2.6)	12 (18.2)	
Not applicable	8 (20.5)	11 (16.7)	
Days to return to work			<0.001
Median (IQR)	11 (7, 14)	14 (14, 21)	
Min–Max	5, 21	7, 30	
Days to return to work, (mean, SD)	12 (5)	16 (6)	0.090

SD stands for standard deviation and IQR for inter-quartile range

### Costs

The base case results show that LA was slightly more expensive compared to OA. The mean overall healthcare cost for LA was $355 (SD: 234) or 147,562 Naira (SD: 97,130) compared to $273 (SD: 213) or 113,556 Naira (SD: 88,559) for OA. The difference in costs between the two groups was $82 (34,006 Naira) in favor of OA. The results appear to be driven by the cost of laparoscopic surgery $312 (SD: 221) or 129,569 Naira (SD: 91,832) compared to $179 (SD: 35) or 74,318 Naira (SD: 14,701) for OA. For reference, the unit costs for individual points of care are listed in Supplementary Table 1. However, the results show that hospital stay, diagnostics, outpatient care and reoperation costs were lower among patients who had LA compared to OA. For example, the hospital stay cost was $11 (SD: 9) or 4,623 Naira (SD: 3,832) in the laparoscopic group compared to $47 (SD: 205) or 19,371 Naira (SD: 85,104) in the OA group. The costs that are associated with SSI for diagnostic, outpatients, and reoperation had a significant impact on the postoperative costs for open surgery. The rest of the base case results are presented in Table 4.

**Table 4:** Base case costs of laparoscopic versus open appendicectomy

Direct costs	Laparoscopic appendectomy (*n* = 39)		Open appendectomy (*n* = 66)		Difference in costs		*p* value
	Mean in Naira (SD)	Mean in $ (SD)	Mean in Naira (SD)	Mean in $ (SD)	Naira	Dollars	
Surgery	129,569 (91,832)	312 (221)	74,318 (14,701)	179 (35)	55,251	133	<0.001
Hospital stay	4,623 (3,832)	11 (9)	19,371 (85,104)	47 (205)	−14,748	−35	0.283
Postoperative costs*	13,371 (6,630)	32 (16)	19,867 (14,039)	48 (34)	−6496	−16	0.008
Diagnostics	11,284 (6,284)	27 (15)	14,221 (6,959)	34 (17)	−2937	−7	0.033
Outpatient costs	2,087 (1,111)	5 (3)	3,442 (3,014)	8 (7)	−1355	−3	0.008
Reoperation**	0 (0)	0 (0)	2,204 (12,370)	5 (30)	−2204	−5	0.269
Revised postoperative***	13,869 (6,948)	33 (17)	23,174 (16,516)	56 (40)	−9304	−22	0.001
**Total direct costs**	147,562 (97,130)	355 (234)	113,556 (88,559)	273 (213)	34,006	82	0.070
**Revised total direct cost**	148,061 (96,850)	356 (233)	116,863 (90,715)	281 (218)	31,198	75	0.100
Lost income	24,605 (10,035)	59 (24)	34,382 (12,567)	83 (30)	−9777	−24	<0.001

SD stands for standard deviation. $ stands for US dollars

*Postoperative costs include: diagnostics, outpatient, and reoperations costs

**Includes readmission

***The revised costs were re-estimated costs after increasing the postoperative healthcare costs by 75.5% when patients developed surgical site infections. Total direct cost include surgery, hospital stay, and postoperative costs

There were more societal benefits associated with quicker return to work among LA patients. LA patients returned to work earlier, as such they lost less income due to missed work $59 (SD: 24) or 24,608 Naira (SD: 10,036) compared to $83 (SD: 30) or 34,386 Naira (SD: 12,568) for OA patients (Table 5). We did not see any change in the results when the discount rate was varied. An increase in either discount rate from 3 to 5% did not change the results as the difference in costs between the two groups remained at $82 (34,006 Naira). Results from the regression analysis show that costs of LA were $14 higher compared to OA, however, the results were not statistically significant (*p* value 0.53). Only age had a significant impact on the overall costs of the patients (Table 6). An increase in age by one year was associated with a significant decrease in costs by $3 (*p* value 0.03).

**Table 5:** Sensitivity analysis results

Resource use	Laparoscopic appendectomy (*n* = 39)		Open appendectomy (*n* = 66)		Difference in costs		*p* value
	Mean in Naira (SD)	Mean in $ (SD)	Mean in Naira (SD)	Mean in $ (SD)	Naira	Dollars	
Useful life at 40 years	146,190 (95,819)	352 (231)	113,395 (88,555)	273 (213)	32,795	79	0.078
Discount rate 5%	147,563 (97,130)	355 (234)	113,556 (88,559)	273 (213)	34,006	82	0.070

SD stands for standard deviation. All costs are in US dollars

**Table 6:** Multivariable linear regression looking at the cost of surgery

Regressors for cost of surgery	Coefficient (US $)	Standard error	95% confidence interval
*Approach*			
Open	–	–	(−29, 57)
Laparoscopic	14	22	
*Sex*			
Female	–	–	(−58, 29)
Male	−15	22	
*Employed*			
No	–	–	(−38, 133)
Yes	48	43	
*Complex histology*			
No	–	–	(36, 55)
Yes	9	23	
*Age in years*	−3	1	(−6, 0)
(Intercept)	329	31	(266, 391)

## Discussion

Based on the estimated healthcare costs alone, LA was slightly more costly compared to OA which appears to be due to the higher cost of the laparoscopic surgery. Further analysis showed that LA patients had lower SSI costs and returned quicker to work compared to open appendicectomy, which made the overall costs between the two groups comparable. Thus, despite having slightly higher healthcare costs, LA had more societal benefits compared to OA. This is particularly important in an LMIC setting like Nigeria where additional cost and periods of return to hospital for wound care can significantly impact households.

Many studies highlighting the reasons for the low adoption of the laparoscopic approach listed cost of laparoscopy, lack of training, challenges of consumable supplies and socio-cultural issues among others. Of these, high cost of initial set-up was top of the list in many studies [4, 20], but the cost of laparoscopy has not been robustly studied and the argument for cost with policy makers has been challenging. The findings in the current study are similar to the findings from a cost-effectiveness analysis in the USA that showed that LA was more costly compared to OA ($6118 vs. $5171) when only healthcare costs were considered but had less cost compared to OA when societal costs were considered ($10,400 vs. $12,055) [21]. However, our results were contrary to that of a retrospective study from Italy [22]. The study found that the cost of LA was slightly lower compared to OA (Euro 2282 vs. Euro 2337) but the difference was not statistically significant.

A major strength of this study is the prospectively collected data on health economic analysis of laparoscopic surgery in LMIC for the first time. This strategy considers the benefit for societies in LMIC of initially investing the money required to increase the number of appendectomies done by laparoscopy. This study has limitations that are important to be acknowledged. First, there was a selection bias of patients included, as the study was performed during the SARS-CoV-2 pandemic. Many patients with uncomplicated acute appendicitis were unable to access care in the tertiary hospitals during the period of lockdowns due to travel restrictions with more complicated cases being referred. Another bias is that most laparoscopic patients were operated on by consultants who are paid higher salaries compared to trainees who predominantly conducted OA. This might have influenced the cost of laparoscopic procedures in this study. We did not account for the difference in cost between surgeons with and without laparoscopic expertise and this could have an impact on the overall cost of LA, although it would be unlikely to show a difference favoring the open approach. Further, the societal cost only included productivity losses but did not include other costs outside the healthcare system which might have underestimated the societal costs. Finally, the costs of SSI were based on a cost-of-illness analysis. However, to our knowledge this is the best available data of the costs associated with SSI in LMICs.

Several efforts are ongoing to address the challenges of training in laparoscopic skills with virtual training programs obviating the need for overseas travel [9, 10]. Previous studies from Nigeria have also demonstrated feasibility of sustained laparoscopic services by incorporating local adaptations and improvisations [4, 23]. Such innovative solutions encourage local adoption by providing mentorship for individuals and institutions aiming to establish laparoscopic services while not relying on expensive and often inconsistent foreign volunteers. We are currently proposing a wider study to include federal medical centers, state-owned hospitals and private hospitals across Sub-Saharan Africa. Further studies to address issues limiting the adoption of laparoscopy and other forms of minimal access surgeries will include those deploying site-specific local training across different countries, improving access to supplies and ensuring sustainability, as well as effecting policy changes toward adoption and upscaling of minimal access surgeries in public hospitals. Policy changes in Nigeria will necessarily engage the multiple levels of government including the local state and federal government health ministries and their critical departments and agencies with the findings of this and similar studies to influence policies on surgical procedures and healthcare funding in the country.

### Supplementary Information

Below is the link to the electronic supplementary material.

Supplementary file1 (DOCX 3376 kb)
