# Supplementary material for: A Prospective, Observational Cost Comparison of Laparoscopic and Open Appendicectomy in Three Tertiary Hospitals in Nigeria
Source: World J Surg. 2023 Oct 11;47(12):3042–50. doi: 10.1007/s00268-023-07148-5 (PMC10694127; doi:10.1007/s00268-023-07148-5)
Supplement: Supplementary file 1 — Supplementary file1 (DOCX 3376 kb) [file 268_2023_7148_MOESM1_ESM.docx]

**Supplementary Figure 1: Location map of Nigerian hospitals which participated in LION**

**
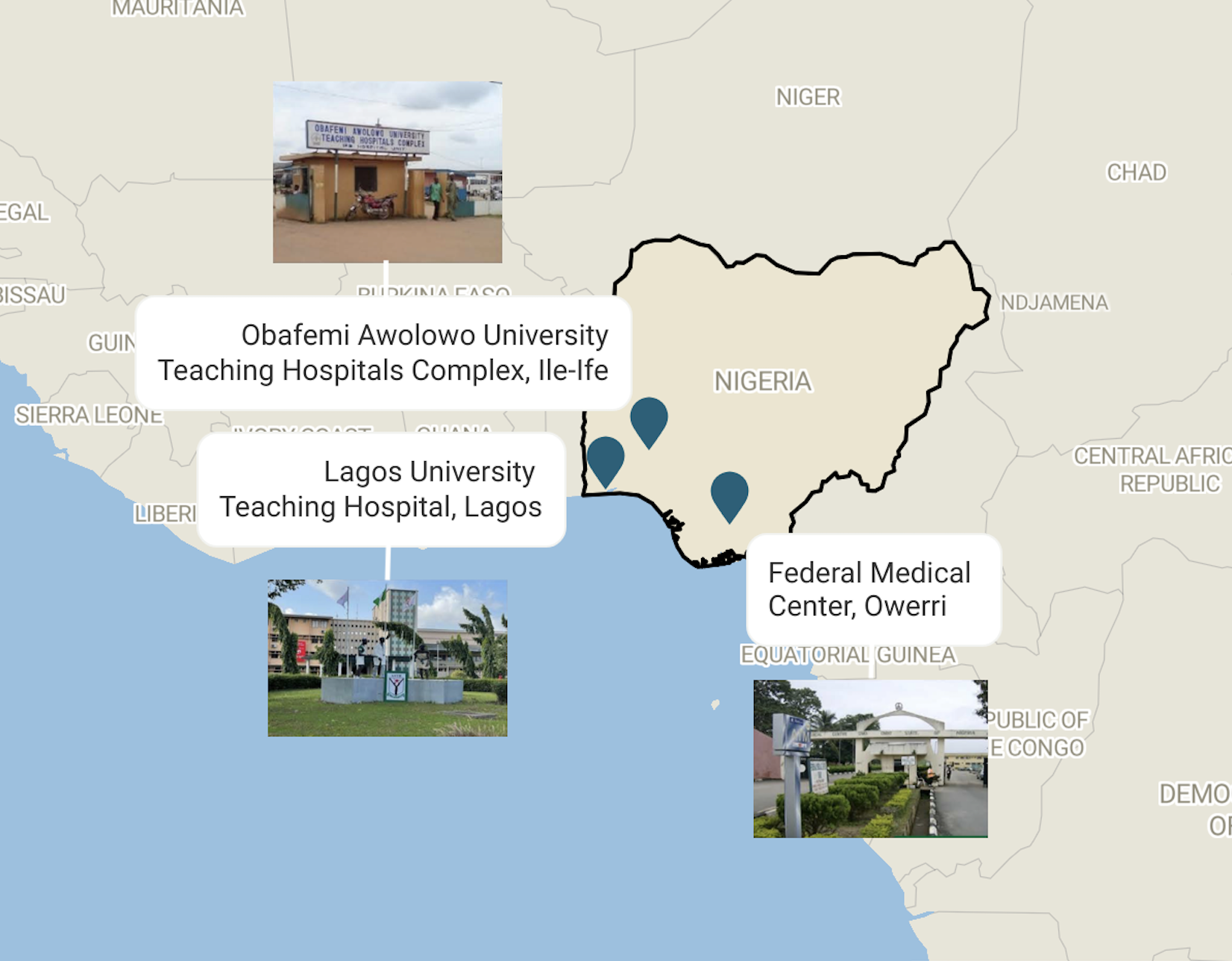
**

**Supplementary Table 1: Unit costs of individual healthcare items and activates**

|  | Nigerian Naira | US dollar |
| --- | --- | --- |
| Estimate cost per visit (if yes to hospital clinic visit) | 1278.51 | 2.78 |
| Estimated cost of transportation to and from consultation | 959.90 | 2.09 |
| Cost per visit to see health worker (community doctor, nurse) | 600.00 | 1.30 |
| Cost of transport to see health worker | 457.14 | 0.99 |
| Cost per minor surgery pack | 8273.33 | 17.97 |
| Cost per major surgery pack | 22329.52 | 48.51 |
| Cost per laparoscopic surgery pack | 18958.10 | 41.19 |
| Open appendectomy operation fee | 47638.10 | 103.50 |
| Laparoscopic appendectomy operation fee | 53257.14 | 115.70 |
| Anaesthesia fees | 22057.14 | 47.92 |
| Average cost per suture | 1185.71 | 2.58 |
| Skin prep cost | 7152.38 | 15.54 |
| Cost per Endoloop | 11190.48 | 24.31 |
| Cost per Endobag | 16047.62 | 34.86 |
| Cost per disposable camera cover | 4357.14 | 9.47 |
| Disposable camera cost | 2500.00 | 5.43 |
| Cost per urinary catheter | 566.67 | 1.23 |
| Cost per swab (gauze) pack | 599.05 | 1.30 |
| Cost per wound dressing | 749.52 | 1.63 |
| Cost per disposable laparoscopic port used | 20542.86 | 44.63 |
| Cost of CO2 cylinder | 50323.81 | 109.33 |
| CO2 Cost | 1879.46 | 4.08 |
| Cost of suction tubing | 1623.81 | 3.53 |
| Purchase price of instruments used for open surgery | 984952.38 | 2139.85 |
| Estimated working life of the instruments used for open surgery (number of cases) | 851.43 | 1.85 |
| Purchase price of the Laparoscopic tower | 8909523.81 | 19356.35 |
| Average yearly number of laparoscopic procedure per tower | 81.55 | 0.18 |
| Purchase price of the anaesthetic machine | 7442857.14 | 16169.95 |
| Average yearly number of cases per anaesthetic machine | 500.50 | 1.09 |
| Open surgery instruments cost per case | 65.75 | 0.14 |
| Lap tower cost per use | 10094.06 | 21.93 |
| Anaesthetic Machine cost per use | 971.41 | 2.11 |
| Cost per x-ray | 3358.10 | 7.30 |
| Cost per CRP/ WCC/ UEs/ LFTs test (average) | 3236.43 | 7.03 |
| Cost per wound swab | 1900.00 | 4.13 |
| Cost for appendix histology | 5888.48 | 12.79 |
| Bed day fee | 2470.48 | 5.37 |
| Diagnostic test costs | 12685.38 | 31.60 |
| Hospital clinic visit cost | 2819.50 | 6.13 |
| Visit to a healthcare worker cost | 2600.00 | 5.65 |
| Transport costs | 953.81 | 2.07 |
| Outpatient costs | 2999.70 | 6.52 |

**Appendix 1: Case report forms**

**
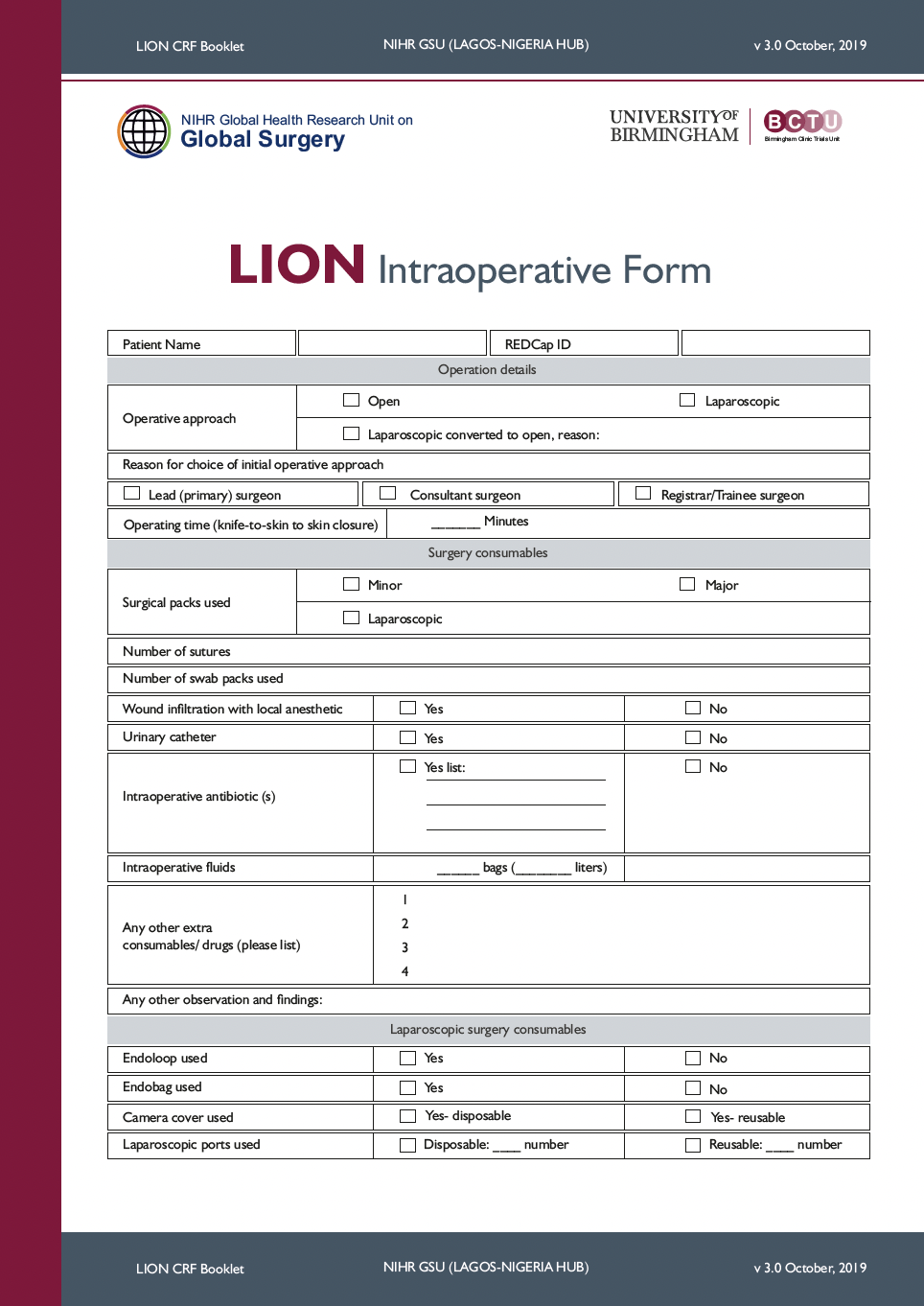
**

**
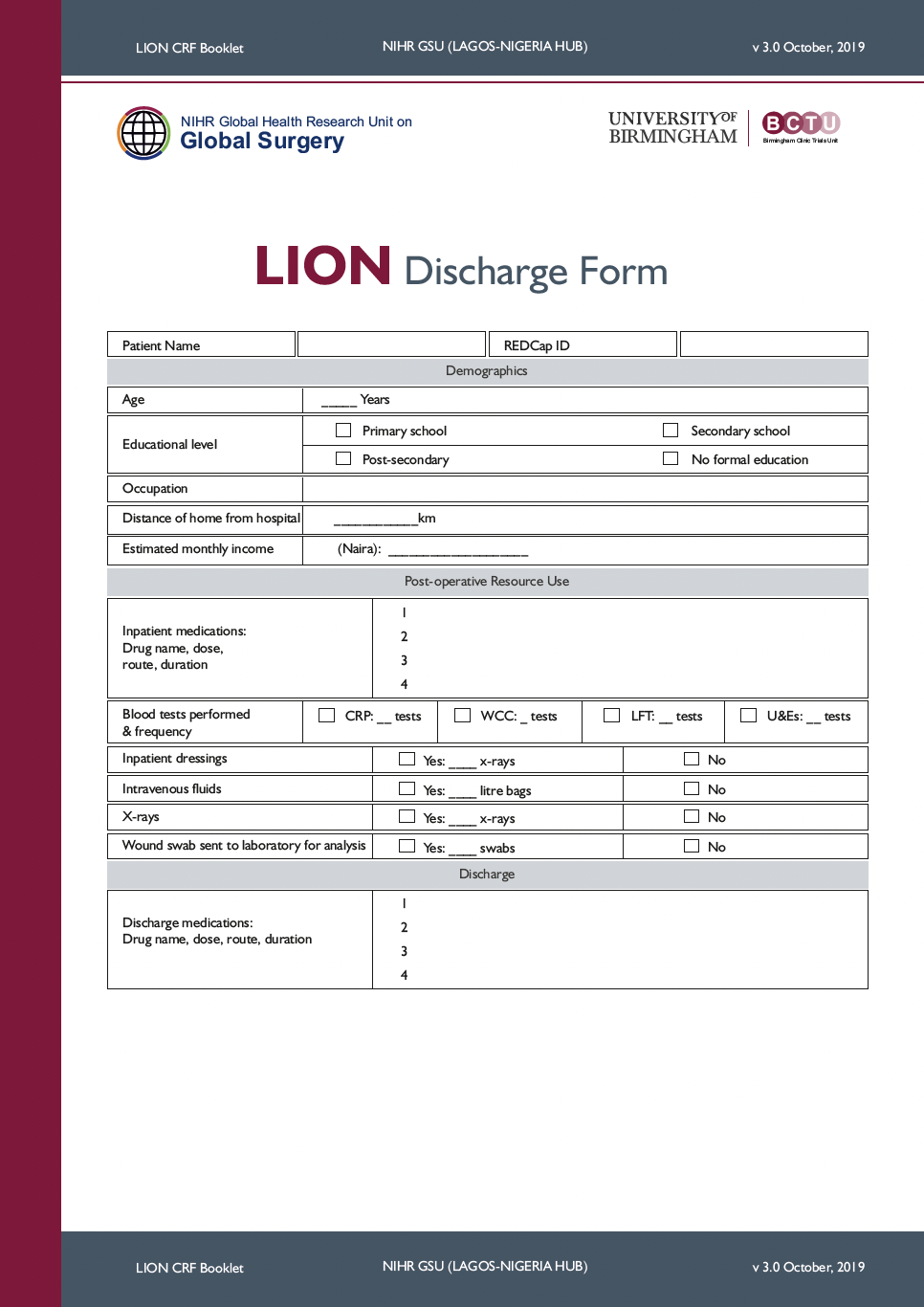
**

**
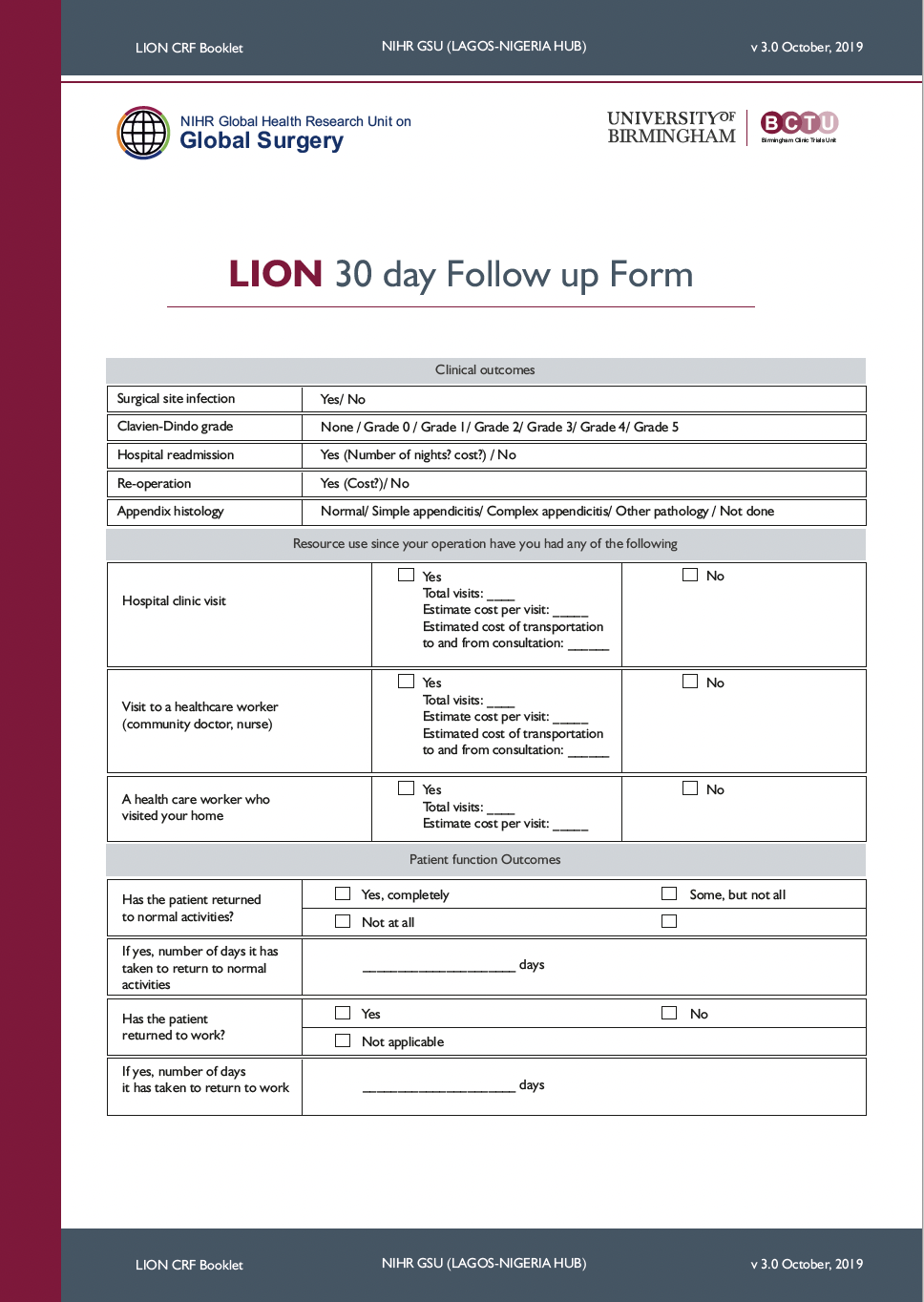
**

**
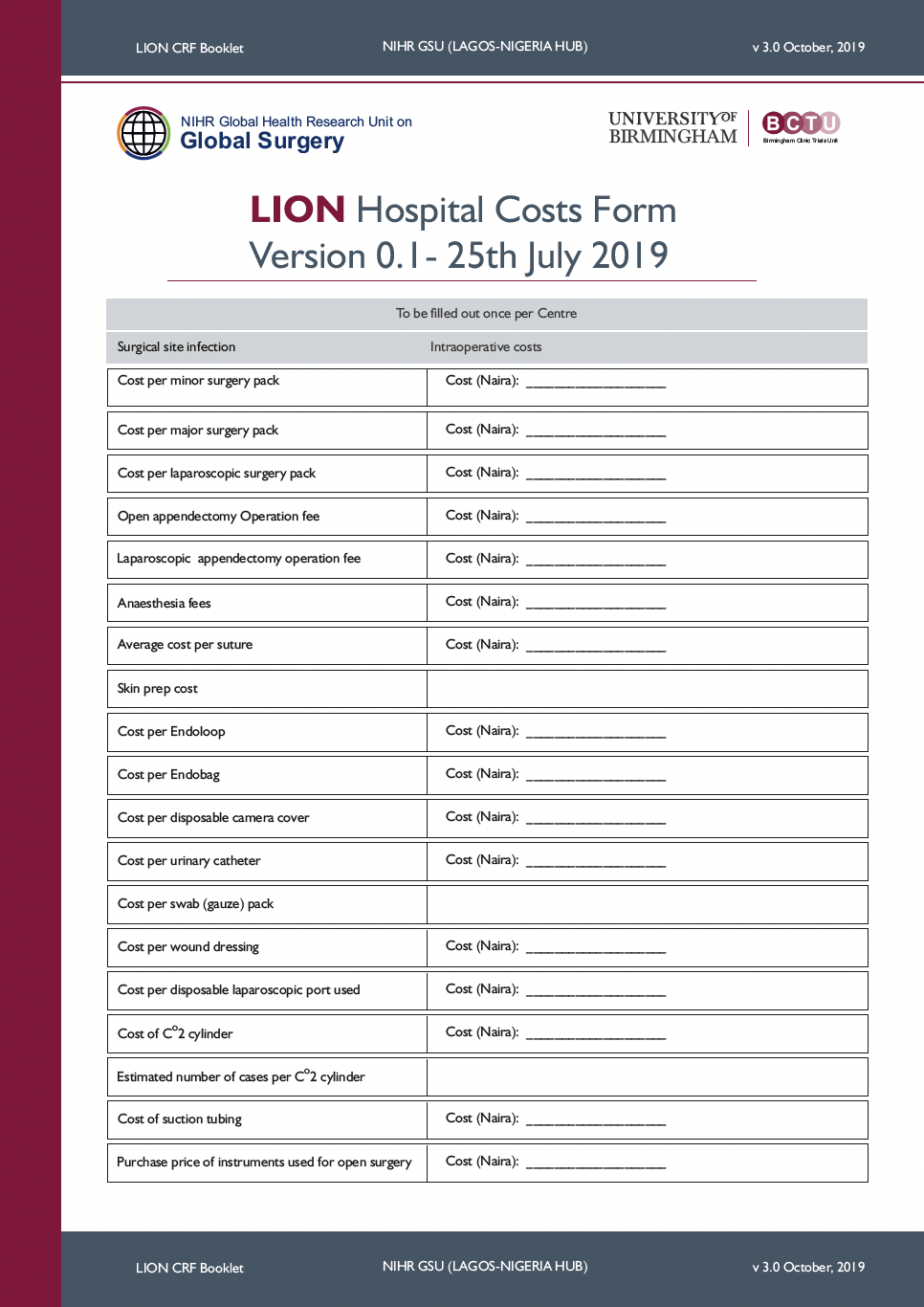
**

**
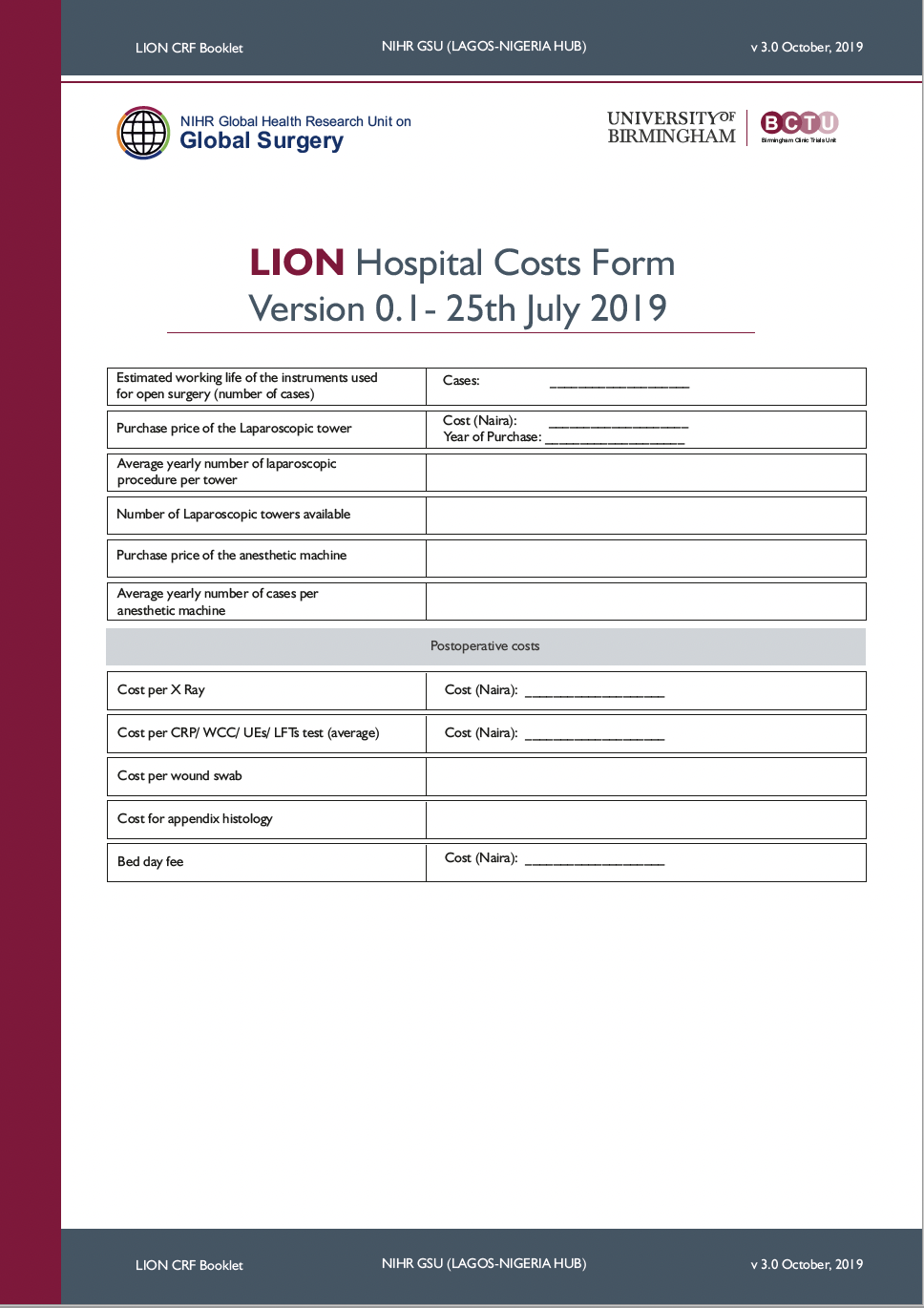
**

**Appendix 2: List of authors**

**Writing committee**

Adewale Adisa^1^, Mwayi Kachapila^2,3^, Christopher Ekwunife^5^_,_ Felix Alakaloko^4^, Balogun Olanrewaju^4^, Bryar Kadir^2^, Dmitri Nepogodiev^2^, Adewale Aderounmu^1^, Innocent Igwilo^5^, Omar Omar^2^, Raymond Oppong^3^, Joana Simoes^2^, Aneel Bhangu^2^, Adesoji Ademuyiwa^4^, on behalf of the NIHR Unit on Global Surgery

*1 Department of Surgery, Obafemi Awolowo University Teaching Hospital, Ile-Ife, Nigeria*

*2 NIHR Global Health Research Unit on Global Surgery, University of Birmingham, Birmingham, UK*

*3 Health Economics Unit, University of Birmingham, Birmingham, UK*

*4 Paediatric Surgery Unit, Department of Surgery, University of Lagos, Lagos, Nigeria*

*5 Department of Surgery, Federal Medical Center, Owerri, Nigeria*

**Writing committee members’ contributions**

Adewale Adisa contributed to the conceptualisation of the study, supervised data collection, wrote the draft and edited the manuscript. Mwayi Kachapila contributed to the designing of data collection forms, conducted the analysis, drafted and edited the manuscript. Ekwunife Christopher, Adesoji Ademuyiwa, Felix Alakaloko, Balogun Olanrewaju, Innocent Igwillo and Adewale Aderounmu, contributed to study design, collected data at the different centers and contributed to manuscript writing. Omar Omar and Bryar Kadir conducted data analysis and edited the manuscript. Raymond Oppong supervised data analysis, edited and commented on the manuscript. Dmitri Nepogodiev and Joana Simoes supported data analysis, manuscript preparation, and manuscript editing. Aneel Bhangu contributed to the conceptualisation of the study, supervised data analysis, wrote the draft and edited the manuscript.
